# SNLI Indo: A recognizing textual entailment dataset in Indonesian derived from the Stanford Natural Language Inference dataset

**DOI:** 10.1016/j.dib.2023.109998

**Published:** 2023-12-21

**Authors:** I Made Suwija Putra, Daniel Siahaan, Ahmad Saikhu

**Affiliations:** a Department of Informatics, Institut Teknologi Sepuluh Nopember, Surabaya, Indonesia; b Department of Information Technology, Udayana University, Bali, Indonesia

**Keywords:** Dataset, Recognizing textual entailment, SNLI, SNLI Indo

## Abstract

Recognizing textual entailment (RTE) is an essential task in natural language processing (NLP). It is the task of determining the inference relationship between text fragments (premise and hypothesis), of which the inference relationship is either entailment (true), contradiction (false), or neutral (undetermined). The most popular approach for RTE is neural networks, which has resulted in the best RTE models. Neural network approaches, in particular deep learning, are data-driven and, consequently, the quantity and quality of the data significantly influences the performance of these approaches. Therefore, we introduce SNLI Indo, a large-scale RTE dataset in the Indonesian language, which was derived from the Stanford Natural Language Inference (SNLI) corpus by translating the original sentence pairs. SNLI is a large-scale dataset that contains premise-hypothesis pairs that were generated using a crowdsourcing framework. The SNLI dataset is comprised of a total of 569,027 sentence pairs with the distribution of sentence pairs as follows: 549,365 pairs for training, 9,840 pairs for model validation, and 9,822 pairs for testing. We translated the original sentence pairs of the SNLI dataset from English to Indonesian using the Google Cloud Translation API. The existence of SNLI Indo addresses the resource gap in the field of NLP for the Indonesian language. Even though large datasets are available in other languages, in particular English, the SNLI Indo dataset enables a more optimal development of deep learning models for RTE in the Indonesian language.

Specifications Table

**Table:** 

Subject	Computer Science
Specific subject area	Natural Language Processing, Recognizing Textual Entailment, Deep learning
Data format	Raw, Filtered
Type of data	Text
Data collection	SNLI Indo is derived from the SNLI corpus, where the premise and hypothesis sentences are translated directly from English to Indonesian using the Google Cloud Translation API. The SNLI corpus is divided into three sets, namely train, development, and test set. The translation process is applied to all the premise and hypothesis sentences in all the three sets. This ensures that the number of sentence pairs obtained is the same as the original SNLI dataset, namely 570k sentence pairs. A filtering process is carried out to remove incomplete sentence pairs and those with a gold label ‘-’. As a result, 569,027 sentence pairs are obtained.
Data source location	SNLI (Stanford Natural Language Inference) https://nlp.stanford.edu/projects/snli/
Data accessibility	Repository name: Mendeley Data Data identification number: 10.17632/k4tjhzs2gd.1 Direct URL to data: https://data.mendeley.com/datasets/k4tjhzs2gd/1

## 1 Value of the Data

• Several studies on RTE in the Indonesian language have introduced Indonesian RTE datasets [1], [2], [3]. However, these datasets consist of fewer than 10,000 sentence pairs. Furthermore, the sentence pairs are acquired through automated techniques or web crawlers. As a result, the naturalness of these sentences is compromised. Additionally, in terms of data quantity, the number of sentence pairs is insufficient for developing reliable and accurate RTE deep learning models.
• The SNLI Indo dataset, that consists of premise and hypothesis sentence pairs in the Indonesian language, enables performance evaluation of RTE models in terms of their ability to understand lexical, syntactic, and semantic features of sentences. For this reason, the SNLI Indo dataset is a valuable resource for Indonesian language NLP research.
• RTE research in various languages has been rapidly evolving and attracting significant attention in recent years due to the necessity of RTE in many NLP tasks and the challenge of developing RTE models that can achieve a high accuracy. Moreover, the introduction of the SNLI corpus in 2015 opened the possibility of carrying out textual entailment recognition using neural networks, which motivated researchers to develop models that can achieve superior performance [4]. The Indonesian language is the tenth morphologically richest language in the world and is spoken by over 270 million people [5]. This reflects the importance and challenging nature of the Indonesian language. Hence, the SNLI Indo dataset can be beneficial in carrying out NLP applications in the Indonesian language that require the task of RTE, such as text summarization, sentiment analysis, information verification, question answering, text classification, and machine translation evaluation [4]. Furthermore, we believe that the proposed SNLI Indo dataset will encourage more research in the field of RTE in the Indonesian language.
• SNLI Indo is an Indonesian language RTE dataset that was constructed by translating the sentence pairs in the existing SNLI dataset from English to Indonesian. The SNLI Indo dataset consists of 549,365 sentence pairs for training, 9,840 sentence pairs for model validation, and 9,822 sentence pairs for testing. SNLI Indo has been used in previous researches [6,7].

## 2 Objective

RTE is an essential task in NLP that determines the inference relationship between two text fragments, namely premise (P) and hypothesis (H), of which the inference relationship is either entailment (true), contradiction (false), or neutral (undetermined). Various approaches have been used to carry out the task of RTE, with neural networks being the most widely adopted approach, in particular deep learning. For deep learning approaches, the availability of large and high-quality RTE datasets is essential in order to achieve high performance. Currently, substantial RTE datasets are available for various languages, including English, Chinese, Italian, Arabic, and Czechoslovakia [4]. As for the Indonesian language, there exists RTE datasets such as INARTE [1] and IndoNLI [3] that contain 500 and 10k sentence pairs, respectively. However, the number of sentence pairs is insufficient for building an optimal RTE deep learning model.

Therefore, the purpose of creating this dataset is to provide a substantial dataset resource for RTE research in the Indonesian language. This initiative aims to accelerate RTE research in the Indonesian language using deep learning approaches, ultimately leading to improved performance of the RTE models. Consequently, it is expected that the resource gap in the field of NLP will be bridged, ensuring that RTE research in the Indonesian language is not left behind compared to RTE research in other languages.

## 3 Data Description

SNLI Indo was created through the process of translating the sentences in the SNLI corpus from English to Indonesian. The SNLI corpus is comprised of approximately 570k sentence pairs [8]. The premise sentences in SNLI are sourced from image captions in the Flickr30k dataset. The hypothesis sentences are manually created through a crowdsourcing framework, in which crowd-workers are tasked to create three corresponding sentences for each premise sentence, one for each label (entailment, contradiction, and neutral). These sentences were then validated by qualified workers.

In the SNLI corpus, each data row has ten columns, which are detailed in Table 1. However, only four columns were translated and included in the SNLI Indo dataset, namely annotator_labels, sentence1, sentence2, and gold_label. Other columns containing sentence parsing structures are considered irrelevant when translated directly into Indonesian. Furthermore, similar to the SNLI corpus, the data in the SNLI Indo dataset is split into three prespecified sets, namely train, development, and test set.

**Table 1:** Dataset column descriptions.

Column	SNLI	SNLI Indo	Description
annotator_labels	√	√	This column contains labels issued by the five annotators.
captionID	√		This column contains image codes found in Flicker30k, whose captions are used as sentence1.
gold_label	√	√	This column contains the final label decided for the relationship between sentence1 and sentence2.
pairID	√		This column contains the connection code of the captionID with the row ID and the given gold label.
sentence1	√	√	This column contains the premise sentence, which is the first sentence in a sentence pair.
sentence1_binary_parse	√		This column contains the binary parse tree of sentence1. The binary parse tree represents the syntactic structure of the sentence.
sentence1_parse	√		This column contains the parse tree of sentence1. The parse tree breaks down the sentence into constituents such as phrases and clauses.
sentence2	√	√	This column contains the hypothesis sentence, which is the second sentence in a sentence pair.
sentence2_binary_parse	√		This column contains the binary parse tree of sentence2. The binary parse tree represents the syntactic structure of the sentence.
sentence2_parse	√		This column contains the parse tree of sentence2. The parse tree breaks down the sentence into constituents such as phrases and clauses.

**Fig. 1:**
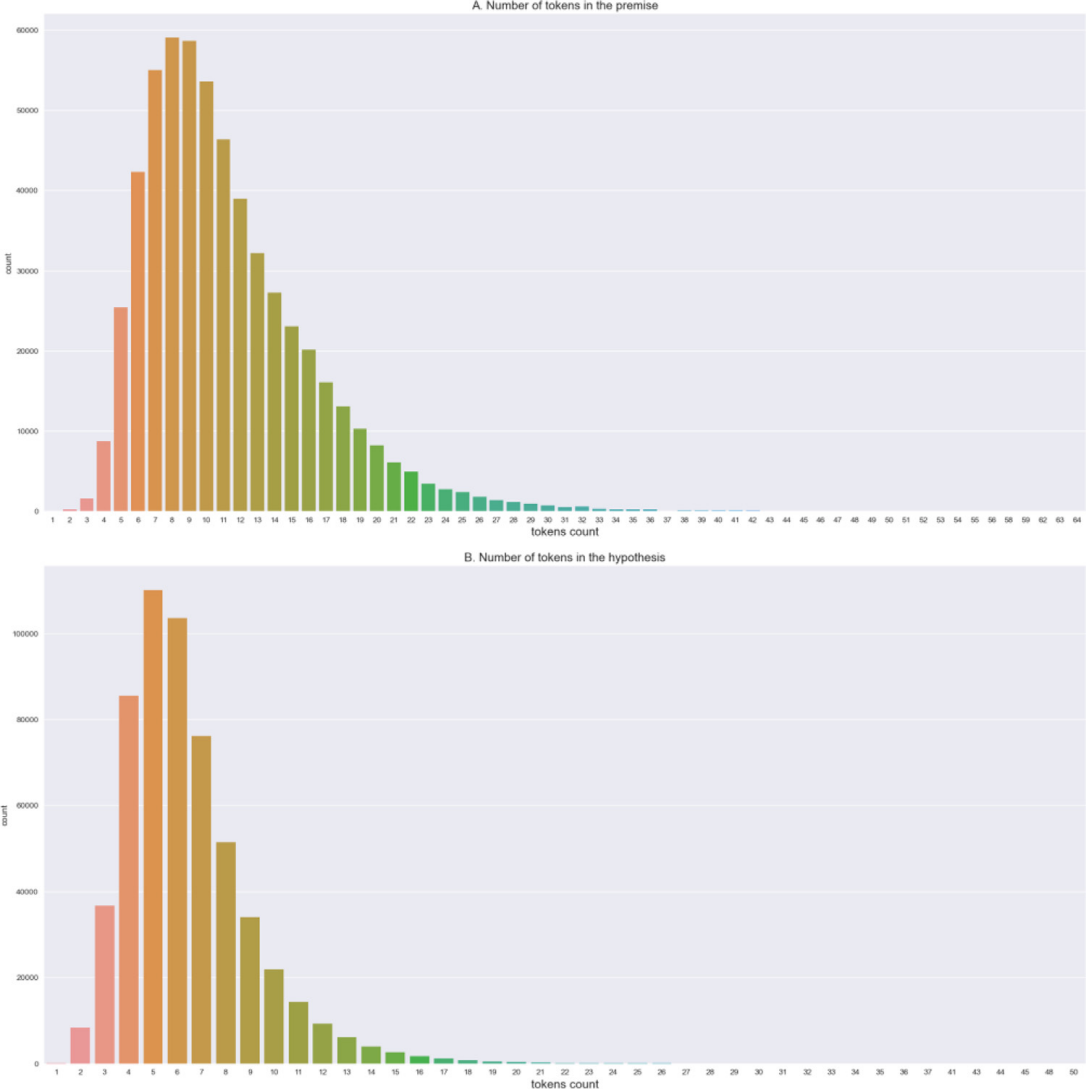
Distribution of sentence lengths based on tokens in the premise (A) and hypothesis (B) sentences.

The statistical comparison between the SNLI corpus and the SNLI Indo dataset can be seen in Table 2. SNLI Indo has fewer sentences in the train set, development set, and test set compared to the SNLI corpus. The is due to the exclusion of sentence pairs in the SNLI corpus that did not have a gold label or were labelled as '-', and also sentences without pairs. These sentence pairs were excluded because they serve no purpose in RTE model development. Regarding the number of tokens in the premise and hypothesis sentences, there are fewer tokens in the SNLI Indo dataset compared to the SNLI corpus. The distribution of sentence length based on token count of the train set of the SNLI Indo dataset is shown in Fig. 1. The x-axis represents the number of tokens in a sentence, and the y-axis represents the number of sentences that have the same number of tokens as indicated by the x-axis.

**Table 2:** Key statistics of the SNLI Corpus and SNLI Indo dataset.

Key Statistics	SNLI	SNLI Indo
Number of sentence pairs in train set	550,152	549,365
Number of sentence pairs in development set	10,000	9840
Number of sentences pairs in test set	10,000	9822
Premise mean token count	12.8	12.1
Hypothesis mean token count	7.4	6.5

**Fig. 2:**
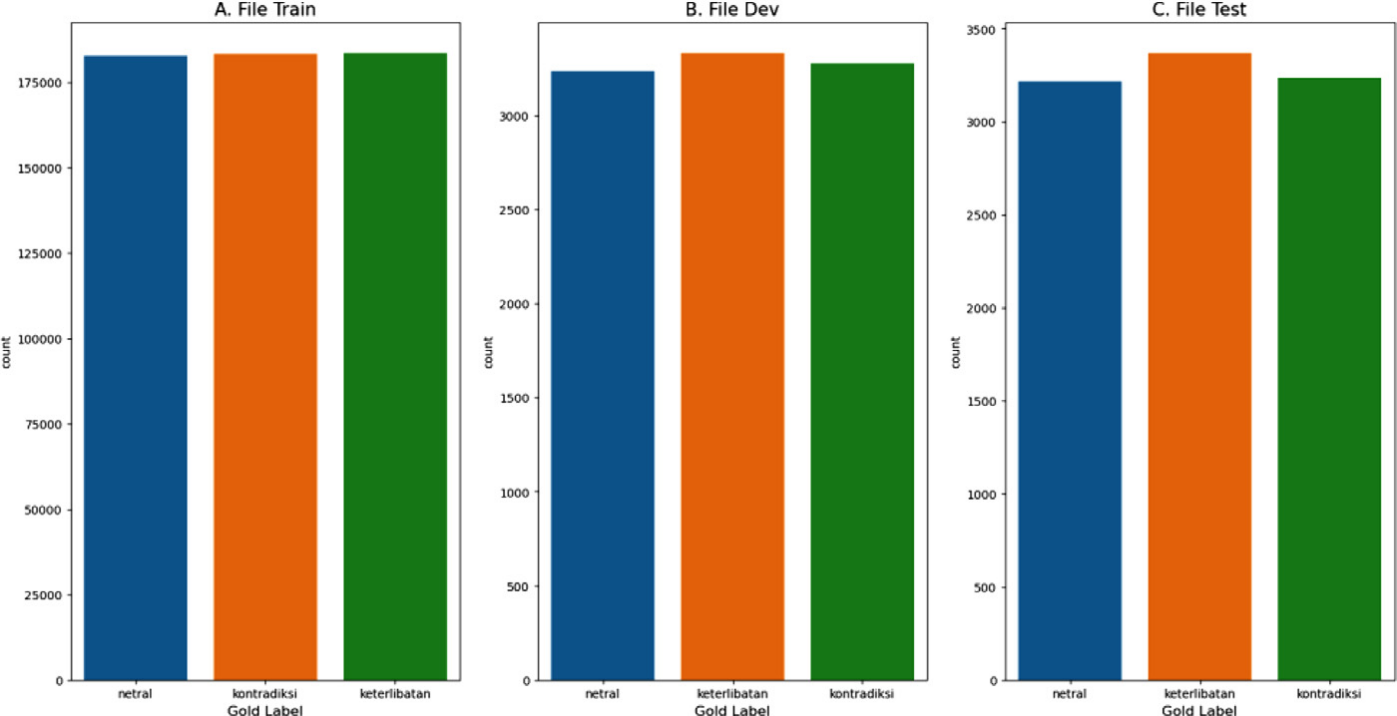
Comparison of labels in the train set (A), dev set (B), and test set (C) of the SNLI Indo dataset (the label “keterlibatan” corresponds to entailment, “kontradiksi” corresponds to contradiction, and “netral” corresponds to neutral).

Distribution of label count is also crucial in a dataset to avoid bias and overfitting [9]. The SNLI Indo dataset has a balanced label distribution for the train set, development set, and test set, as shown in Fig. 2. This is evident from the similar heights of the bar graphs for each label in each set.

**Fig. 3:**
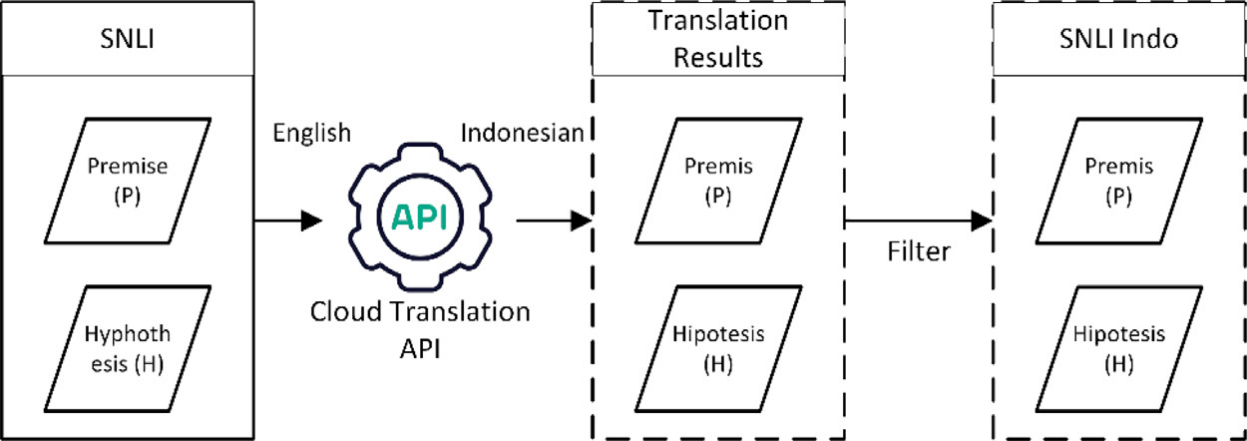
The flow of the translation process for building the SNLI Indo dataset from the SNLI Corpus.

Each premise sentence (sentence1) and hypothesis sentence (sentence2) in the SNLI corpus were directly translated from English to Indonesian using the Google Cloud Translation API tool to create the sentence pairs in the SNLI Indo dataset. The flow of the language translation process is illustrated in Fig. 3.

Translation was carried out on all the premise and hypothesis sentences in the train, development, and test sets of the SNLI corpus. Consequently, the translation process resulted in the same number of sentence pairs as the original SNLI corpus. The translation process was followed by a filtering process. The filtering process involved excluding sentence pairs that were incomplete and had a gold label of '-'. The sentence pairs that passed the filtering process were all included into the SNLI Indo dataset and were also grouped into three sets, namely train, development, and test set, similar to that of the SNLI corpus. The resulting number of sentence pairs that are present in the SNLI Indo dataset is sufficient to be used for testing RTE models in solving RTE tasks in Indonesian.

The Google Cloud Translation API, which was developed by Google, was utilized in this research as the automatic translation tool because it has been proven to be adequately accurate in intelligent machine translation. The Google Cloud Translation API remains a reliable tool for translating simple sentences using standard and coherent phrase levels [10,11].

Table 3 shows several examples of sentence pairs in the SNLI corpus and their translation into Indonesian in the SNLI Indo dataset. The columns SNLI Premise and SNLI Hypothesis contain the original English sentence pairs from the SNLI corpus [8]. Meanwhile, the columns SNLI Indo Premise and SNLI Indo Hypothesis are the results of translation of the sentence pairs into Indonesian.

**Table 3:** Four examples of sentence pairs from the SNLI corpus and their translations in Indonesian.

SNLI Premise	SNLI Hypothesis	SNLI Indo Premise	SNLI Indo Hypothesis	Label
a large, middle-age women in a red dress with white polka dots, singing at a microphone.	a skinny girl in a bathing suit.	seorang wanita paruh baya besar dalam gaun merah dengan bintik-bintik putih, bernyanyi di depan mikrofon.	seorang gadis kurus dalam pakaian renang.	contradiction
three boys are playing soccer.	boys are playing.	tiga anak laki-laki sedang bermain sepak bola.	anak laki-laki sedang bermain.	entailment
a shot of bicyclists in a race with the background blurred by their speed.	bicyclists moving so quickly trying to beat each other to the finish line.	bidikan pengendara sepeda dalam perlombaan dengan latar belakang diburamkan oleh kecepatan mereka.	pengendara sepeda bergerak begitu cepat mencoba untuk saling mengalahkan hingga garis finis	neutral
a man is giving instructions to several children in a forest.	a person is giving instructions to several children in a forest.	seorang pria sedang memberikan instruksi kepada beberapa anak di hutan.	seseorang sedang memberikan instruksi kepada beberapa anak di hutan.	entailment

The first sentence pair in Table 3 is an example of a sentence pair that possesses a nested clause in the premise sentence and a hypothesis sentence in which all the words differ to those of the premise sentence. Therefore, lexically and semantically, this sentence pair is labeled as a contradiction. The second sentence pair is an example of a pair in which both the premise and hypothesis have short sentence forms, indicating an entailment relationship. The third sentence pair contains a hypothesis that explains the object in the premise sentence, indicating that the inference relationship between the sentences is neutral. Meanwhile, the sentence pair in the last row is an example where the premise and hypothesis have the same number of tokens. Based on these four examples, the original sentence pairs and the translated sentence pairs possess the same semantic meaning.

## 4 Experimental Design, Materials and Methods

The sentence pairs in the SNLI Indo dataset, which are the results of the translation process, need to be validated. Validation of the translated sentence pairs was carried out to assess the quality of the SNLI Indo dataset. If the validation results are good, then SNLI Indo can be used as a valuable resource in future research on RTE in Indonesian.

**Fig. 4:**
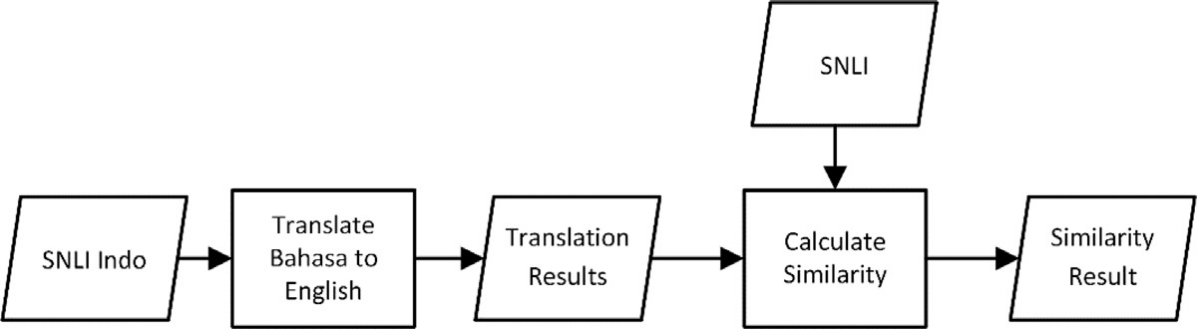
The flow of the validation process using the similarity calculation methods.

The phases of the validation process can be seen in Fig. 4. The sentence pairs in SNLI Indo were retranslated back to English using the same tool. The translated results were then compared to the original sentence pairs in the SNLI corpus to calculate their similarity. Jaccard Similarity and Cosine Similarity were used as the methods for similarity calculation in this research. The output of the validation process is the percentage of similarity between the original and retranslated sentences.

In the validation process, we employed data sampling techniques to obtain a representative subset of sentence pairs for similarity calculation. Data sampling was performed randomly on 1% of the total data, which amounts to 5700 sentence pairs. The sampling technique that was employed involves using the ‘sample()’ function from the Pandas library, which generates a random sample of n rows. Additionally, our random technique utilizes the random function from the Pandas library to extract data randomly from a Pandas data frame.

Table 4 shows the level of similarity between the retranslated and original premise and hypothesis sentences that was calculated using Jaccard similarity and Cosine similarity. The percentage of similarity obtained using Jaccard similarity is lower compared to the percentage of similarity obtained using Cosine similarity for both the premise and hypothesis sentences with a difference of 13% and 11%, respectively. The difference in the percentage of similarity can be attributed to the characteristic of the Jaccard Similarity method, which only considers surface-level or lexical word features in the sentences during its calculation [12]. Meanwhile, Cosine similarity calculates the percentage of similarity by taking into account other features other than lexical features, namely syntactic and semantic features. This is the reason why we used both methods for calculating similarity between the retranslated and original sentences.

**Table 4:** The calculation results indicating the level of similarity between the original and retranslated premise and hypothesis sentences.

Jaccard Similarity		Cosine Similarity	
Premise	Hypothesis	Premise	Hypothesis
66%	69%	79%	80%

The highest percentage of similarity obtained was 79% for the premise sentences and 80% for the hypothesis sentences. These values fall within the "Red" range (75-100%) of the Turnitin standard percentage range. This range signifies the highest level of similarity. These results indicate that the translation process from English to Indonesian did not significantly alter the lexical structure of the sentences and the meaning of the sentences was also preserved. However, validation by measuring the similarity level between the retranslated sentences and the original sentences is not adequate enough as mistakes in the Indonesian sentences can be corrected or reverted by the machine translation tool when translating the Indonesian sentences back to English, thus mistakes in the Indonesian sentences can be overlooked in the validation process.

Therefore, we applied three quantitative metrics to further validate the quality of the sentences in the SNLI Indo dataset, namely BLEU (Bilingual Evaluation Understudy), METEOR (Metric for Evaluation of Translation with Explicit Ordering), and GLUE (General Language Understanding Evaluation). All three metrics were originally designed to evaluate NLP systems, in particular for evaluating the results of machine translation. These metrics perform evaluation at the lexical level. To obtain the metric scores, machine-generated translations, referred to as “candidates,” are compared to human-generated translations, referred to as “references”. These quantitative metrics provide further assurance of the quality of the sentences within the SNLI dataset and can be used to indicate that the SNLI Indo translation mechanism is unbiased.

BLEU is a quality metric based on a weighted average of matching n-grams (sequences of n words) between the candidate and reference translations [13]. For each n-gram sequence, its precision is calculated by counting the number of n-grams in the candidate sentence that occur in the reference sentences and dividing the total matching count by the total number of n-grams in the candidate sentence, while taking into consideration the maximum occurrence of the words in the reference translations. BLEU uses a brevity penalty to adjust the final metric score for candidate translations that are shorter than the reference translations [14]. Similar to BLEU, METEOR is used for evaluating machine translations. METEOR also employs precision, recall, and brevity penalty techniques in matching unigrams to evaluate translation results [15]. Matching in METEOR takes into account synonymous words and also morphological variations. This makes it more robust against synonyms and paraphrases compared to BLEU [16]. Meanwhile, GLUE is a metric that shares similarities with BLEU, but has different minimum values for recall and precision as well as a distinct brevity penalty function. The goal is to better account for sentence length when evaluating the performance of machine translation systems. GLUE combines exponential average precision at different n-gram levels, giving more attention to n-grams with the highest order. This can be beneficial in capturing the quality of phrases in both longer and shorter sentences [17].

**Fig. 5:**
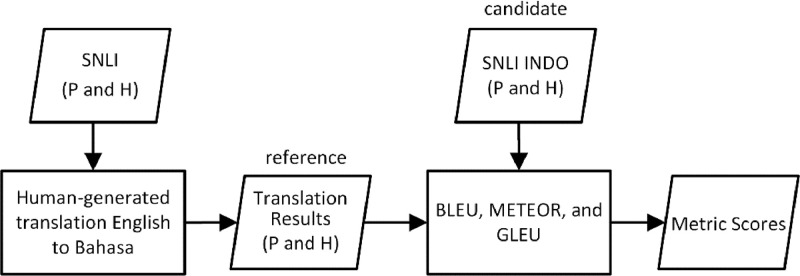
The flow of the validation process using the quantitative metrics

The phases of the validation process using the quantitative metrics can be seen in Fig. 5. Each English premise and hypothesis sentence in the original SNLI corpus is first translated into Indonesian manually by language experts. The human-translated sentences who serve as the reference translations. The Indonesian premise and hypothesis sentences in the SNLI Indo dataset are positioned as the candidate translations. Following a pre-processing step, the candidate translations are then evaluated against the reference translations using BLEU, METEOR, and GLUE metrics. The output of this validation process is the metric scores that indicate the quality level of the sentences in the SNLI Indo dataset.

**Fig. 6:**
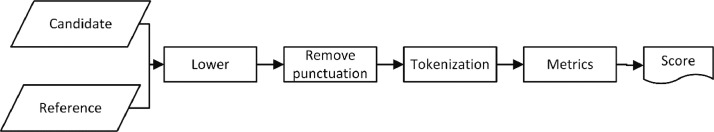
The phases of pre-processing the candidates and references sentences.

We employ several preprocessing steps, as illustrated in Fig. 6, to prepare the candidate and reference sentences before the evaluation process using the quantitative metrics. The initial stage of preprocessing involves converting capital letters to lowercase to standardize the lexical form of the words. Next, we remove punctuation from both the candidate and reference sentences to ensure that punctuation does not affect individual n-grams. The final stage of preprocessing is to tokenize the sentences, extracting the words present in each sentence.

In the implementation, we employed data sampling techniques to obtain a representative subset of sentence pairs for similarity calculation. Data sampling was performed randomly on 0.1% of the total data, resulting in 570 sentence pairs. The sampling technique used in the previous validation using the similarity calculation methods was also used in this validation process. Furthermore, we employed one language expert to produce human-generated translations used as the reference translations. We implemented the BLEU, METEOR, and GLUE metrics using the NLTK library, which is readily available [18], [19], [20].

The metric scores from each of the three metrics fall in the range between 0 to 1, with 0 indicating the lowest level of quality and 1 indicating the highest level of quality. The metric score can be classified into three levels of translation quality. A metric score below 0.30 indicates low translation quality. Scores above 0.30 up to 0.50 generally reflect translation results that are understandable or of medium quality. Scores above 0.50 typically indicate good translation results and are associated with previous translations [21].

Table 5 shows the mean metric scores for the premise sentences and the hypothesis sentences for each quantitative metric. The mean score is obtained by averaging the scores obtained from each candidate-reference pair. A distinct mean score is calculated for the premise sentences and hypothesis sentences. The three metrics exhibit varying metric scores. The BLEU metric, in particular, has a significantly lower score compared to the other two metrics, with a mean score of 0.58 for the premise sentences and 0.55 for the hypothesis sentences. These results can be attributed to fact that BLEU does not take into account synonymous words and morphological variations [14]. In addition, BLEU does not evaluate the fluency or grammatical correctness of translations. On the other hand, METEOR has the ability to detect synonyms and morphological variations [16], while GLUE calculates n-gram precision over references by giving more weight to n-grams that are correct [17]. This results in both metrics producing metric scores above 0.75. Specifically, GLUE is a metric that can be used not only for validating machine translation but also for measuring language understanding in other NLP task domains. This is due to its capability in Grammatical Error Correction (GEC) [22]. This is one of the main advantages of GLUE, resulting in a mean score of 0.81 for the premise sentences and 0.78 for the hypothesis sentences.

**Table 5:** The resulting scores of the quantitative metrics used for validation.

Metrics	Mean Score Premise (P) Sentence	Mean Score Hypothesis (H) Sentence
BLEU	0.58	0.55
METEOR	0.80	0.75
GLUE	0.81	0.78

The SNLI Indo dataset has hypothesis sentences that tend to be shorter than the premise sentences, as indicated by the statistics in Table 2. This results in the difference in mean score between the premise sentences and the hypothesis sentences. The BLEU metric has a mean score difference of 0.03 between the premise sentences and hypothesis sentence. This may be caused by the fact that BLEU is highly influenced by sentence length and has a predefined maximum n-gram length [14]. Hence, the occurrence of lexically overlapping n-gram pairs will result in a low score, especially in shorter sentences. This is different from the METEOR and GLUE metrics, in which both use the concept of flexible unigram matching. This makes both metrics more sensitive towards sentence length, resulting in a larger score difference between the premise sentences and hypothesis sentences. Regarding the metric scores between the GLUE and METEOR metrics, the mean GLUE score for the hypothesis sentences was higher by 0.03 compared to the mean METEOR score. While the mean GLUE score for the premise sentences was only higher by 0.01 than the mean METEOR score. This is because GLUE is still able to adequately assess even when short reference sentences are often encountered for the candidate hypothesis sentences. Based on the overall validation results, the metric scores obtained using the three different evaluation metrics indicate that the quality level of the translated sentences in the SNLI Indo dataset is acceptably high. Therefore, it can be considered as an alternative dataset resource for RTE research in Indonesian.

The SNLI Indo dataset has been utilized in prior research to develop state-of-the-art Indonesian language RTE models [6,7]. In this research, experiments were conducted using neural network approaches, specifically two types of Recurrent Neural Networks (RNN), namely Long Short-Term Memory (LSTM) and Bidirectional (BiLSTM) networks [23]. The basis of the network architecture is a representation-based framework in which the encoding of the premise and hypothesis sentences is carried out separately and the prediction of inference relationship is based on the fused encoded results using the Softmax activation function. Both models were trained using a dataset size of 5,000. Furthermore, the Glove 50-dimensional word embedding technique was employed in both models [24]. We also defined specific parameters in the model training process, including 20 for the number of epochs, 0.001 for the initial learning rate, 128 for the batch size, and 1 for the verbose parameter. The experimental results can be observed in Table 6. The highest accuracy of 73.95% was obtained by the BiLSTM model.

**Table 6:** Performance of deep learning models on SNLI Indo.

Model	#Parameter	Train_accuracy	Val_accuracy
LSTM with Glove Word Embedding 50D	2.3 M	67.25	49.70
BilSTM with Glove Word Embedding 50D	8,5	73.95	48.00

From the validation process that was conducted on the premise and hypothesis sentences in the SNLI Indo dataset, it can be concluded that the translated sentences possess similar lexical forms and semantic meaning to that of the original sentences. Therefore, SNLI Indo can be used as a dataset resource in future research on Indonesian RTE.

## Limitations

Not applicable.

## Ethics Statement

No human or animal studies were conducted in this research. We anonymized all content from social media pages, and no records of personal information were kept.

## CRediT authorship contribution statement

**I Made Suwija Putra:** Software, Formal analysis, Resources, Data curation, Writing – original draft. **Daniel Siahaan:** Conceptualization, Methodology, Validation, Writing – review & editing, Supervision, Project administration, Funding acquisition. **Ahmad Saikhu:** Methodology, Investigation, Validation, Visualization, Supervision, Writing – review & editing.

## Data Availability

SNLI Indo : Dataset Recognizing Textual Entailment (RTE) (Original data) (Mendeley Data)
